# Quality of life assessment of patients with and without venous ulcer^1^

**DOI:** 10.1590/0104-1169.3304.2454

**Published:** 2014

**Authors:** Thalyne Yurí Araújo Farias Dias, Isabelle Katherinne Fernandes Costa, Márjorie Dantas Medeiros Melo, Sandra Maria da Solidade Gomes Simões de Oliveira Torres, Eulália Maria Chaves Maia, Gilson de Vasconcelos Torres

**Affiliations:** 2 Doctoral student, Universidade Federal do Rio Grande do Norte, Natal, RN, Brazil; 3 PhD, Adjunct Professor, Departamento de Enfermagem, Centro de Ciências da Saúde, Universidade Federal do Rio Grande do Norte, Natal, RN, Brazil; 4 Undergraduate student in Nursing, Departamento de Enfermagem, Centro de Ciências da Saúde, Universidade Federal do Rio Grande do Norte, Natal, RN, Brazil; 5 PhD, Associate Professor, Departamento de Psicologia, Centro de Ciências Humanas Letras e Artes, Universidade Federal do Rio Grande do Norte, Natal, RN, Brazil; 6 PhD, Full Professor, Departamento de Enfermagem, Centro de Ciências da Saúde, Universidade Federal do Rio Grande do Norte, Natal, RN, Brazil

**Keywords:** Quality of Life, Varicose Ulcer, Leg Ulcer, Venous Insufficiency, Nursing

## Abstract

**OBJECTIVES:**

to compare the quality of life of patients with chronic venous disease with and without ulcer and to identify the most affected aspects.

**METHOD:**

cross-sectional study with a sample of 204 patients with chronic venous disease. The quality of life was assessed with the help of the SF-36 questionnaire. To compare the scores between the groups, the Mann-Whitney test was used, considering a statistically significant difference when p<0.05.

**RESULTS:**

the quality of life score of patients with ulcer was lower when compared to that of patients without ulcer, in all domains and dimensions of the SF-36, particularly in the domains physical aspect and functional capacity, with very low scores.

**CONCLUSION:**

all aspects of quality of life were more compromised in people with ulcers. These findings can contribute towards a better understanding of the effects of chronic venous disease on the quality of life and towards a better orientation of therapeutic interventions in this population.

## Introduction

Chronic venous disease (CVD) is provoked by valve incompetence or obstruction with interruption of the venous return blood flow in the deep veins of the lower limbs, which causes venous hypertension and compromises the blood irrigation of the tissues in the affected limb, which can lead to the appearance of a venous ulcer (VU)^(^ 1 ^)^.

The clinical manifestations deriving from CVD can be classified based on the classification Clinical manifestations, Etiologic factors, Anatomic distribution of disease, Pathophysiologic findings (CEAP). According to that classification, the clinical signs are categorized in seven classes: Class C0 - non-visible and non-palpable signs of venous disease; Class C1 - telangiectasies or spider veins; Class C2 - varicose veins; Class C3 -edema; Class C4 - alterations of skin and subcutaneous tissue due to the venous disease (4a - pigmentation or eczema and 4b - lipodermatosclerosis or white atrophy); Class C5 - skin alterations with healed ulcer and Class C6 - skin alterations with active ulcer^(^ 2 ^)^.

In Brazil, VU are a serious public health problem, due to the large number of patients with altered skin integrity, although care records in this respect are scarce. The large number of patients with venous ulcers contributes to increase public spending in the Unified Health System (SUS) and interferes in the population's quality of life (QoL) due to complications that can result in significant morbidity^(^ 3 ^-^ 5 ^)^.

Different factors, such as pain, mobility difficulties, reduced self-esteem, social isolation, disability to work, altered body image and depression affect the QoL of people with chronic lower limb injuries. These patients need holistic and multiprofessional care, besides facilitated access to health services^(^ 6 ^)^. A careful and precise assessment of people with VU is essential to guarantee timely and appropriate treatment.

Some authors^(^ 3 ^,^ 7 ^)^ compared the QoL of patients with mild (CEAP= 1, 2 and 3) and severe venous disease (CEAP= 4, 5 and 6), but no studies were found that compared the QoL of patients with ulcers (CEAP=6) with that of patients without ulcers or with healed ulcers (CEAP=1, 2, 3, 4 and 5). The objectives in this study were to compare the quality of life of patients with chronic venous disease with and without venous ulcer and to identify the most affected aspects.

## Method

A comparative and cross-sectional study with a quantitative approach was developed between October 2011 and July 2012. The non-probabilistic sample consisted of 204 CVD patients attended by an angiologist, who were classified regarding the presence or not of venous ulcers based on the clinical CEAP, with group 1 including CEAP 6 (active ulcer) and group 2 CEAP 1 to 5 patients (presence of CVD signals without ulcer).

The study was undertaken at the angiology outpatient clinic of a University Hospital in Natal - RN. Patients who attended to the following inclusion criteria were selected to participate in the study: signs of chronic venous disease; age over 18 years; being attended at the referral hospital and possessing cognitive conditions to answer the research instruments. Patients with arterial or mixed ulcers were excluded from the study. Before their inclusion, the patients received information about the study objectives and those who accepted to participate signed the Informed Consent Form.

Two data collection instruments were used: a structured interview form with sociodemographic and health characteristics and the health-related quality of life (HRQoL) questionnaire SF-36^(^ 8 ^)^. All the study participants answered the sociodemographic form and the SF-36 through an interview with the researcher in a calm and private environment.

The sociodemographic and health variables were: sex, age, marital status, education, income, occupation, chronic illnesses and sleep. The care characteristics were: use of compressive therapy, orientations, laboratory and specific tests, referral and counterreferral and documentation of clinical findings.

The SF-36 é is a multidimensional questionnaire that has been validated in Brazil and is widely used, including 36 items encompassed by eight components: functional capacity (10 items), physical aspects (4 items), pain (2 items), general health status (5 items), vitality (4 items), social aspects (2 items), emotional aspects (3 items) and mental health (5 items), besides one comparative assessment question between the current health conditions and a year earlier. This instrument assesses both the negative (disease) and positive aspects (wellbeing)^(^ 8 ^)^.

The study attended to the ethical principles in the World Medical Association's Helsinki Declaration and complied with Resolution 196/96^(^ 9 ^)^. Next, it was submitted to the Research Ethics Committee and received a favorable opinion (Protocol 279/09).

The collected data were transferred to a database in a Microsoft Excel 2007 worksheet and. After correction, they were exported to and analyzed in statistical software for descriptive analyses with absolute and relative frequencies, means, standard deviations and inferential analysis in the crossing of the variables, with statistical significance set as p≤0.05. The Mann-Whitney Test was applied to check for significant differences between the mean values in the domains and dimensions of the QoL and the investigated patient groups (with and without VU).

## Results

As regards the sociodemographic characteristics, in the study, patients from the state capital (66.2%), female (74.5%), married/fixed partner (63.7%), with a low education level (75.0%), with a profession/occupation (63.7%) and gaining less than one minimum wage (81.9%) were predominant, as displayed in Table 1. The mean age of patients without VU was 53.7 (±13.5), minimum 18 and maximum 86 years. For the patients with VU, the mean age was 60.6 (±11.4), minimum 39 and maximum 92 years.

**Table 1:** Distribution of patients with and without venous ulcer according to sociodemographic and health characteristics, Natal, RN, Brazil

Sociodemographic and health characteristics		Type of patient					p-value
		With venous ulcer			Without venous ulcer		
		n	%		n	%	
Sex							0.054
	Female	69	33.8		83	40.7	
	Male	31	15.2		21	10.3	
Age range							0.009
	As from 60 years	55	27.0		39	19.1	
	Up to 59 years	45	22.1		65	31.9	
Marital status							0.361
	Single/widowed/divorced	38	18.6		36	17.6	
	Married/fixed partner	62	30.4		68	33.3	
Education							0.007
	Up to primary education	83	40.7		70	34.3	
	Secondary and higher education	17	8.3		34	16.7	
Profession/occupation*							0.018
	Present	56	27.5		74	36.3	
	Absent	44	21.6		30	14.7	
Income							0.025
	<1 Minimum wage	76	37.3		91	44.6	
	≥1 Minimum wage	24	11.8		13	6.4	
Chronic Conditions							0.006
	Present	60	29.4		43	21.1	
	Absent	40	19.6		61	29.9	
Sleep							0.314
	<6 hours	25	12.3		22	10.8	
	≥6 hours	75	36.8		82	40.2	
Total		100	49.0		104	51.0	

* domestic servant, driver, cashier, telephone operator, craftsman, cook

† minimum wage in Reais during the study period: R$622; corresponding amount in dollars: U$282.72

The presence of venous ulcers was more frequent among people over 60 years of age (p=0.009), with low education levels (p=0.007), professionally active (p=0.018) and gaining less than one minimum wage (p=0.025). As regards the health characteristics, 50.5% of the subjects suffered from chronic conditions, which were more present in patients with VU (p=0.006). No significant difference was found between with and without venous ulcers and the hours of sleep. Most of the patients assessed (77.7%) slept more than 6 hours/day (Table 1).

The quality of life of patients with and without venous ulcer was different in the eight domains and two dimensions of the SF-36. The mean scores of the patients with VU were lower in all dimensions and domains of the SF-36 when compared to the patients without VU, particularly regarding physical health and functional capacity, with very low mean scores (4.75 and 14.85, respectively). In addition, the low mean scores in the physical health dimension and in the social aspects domain should be highlighted (Table 2).

**Table 2:** Distribution of mean scores in the domains and dimensions of the SF-36 in patients with and without venous ulcer, Natal, RN, Brazil

Quality of life domains and dimensions	With venous ulcer (N=100) Mean (SD*)	Without venous ulcer (N=104) Mean (SD*)	p-value
Functional Capacity	14.85 (20.21)	38.51 (29.40)	<0.001
Physical Aspect	4.75 (16.16)	29.09 (42.97)	<0.001
Pain	33.97 (27.44)	45.83 (23.38)	0.001
General Health Status	36.01 (15.69)	44.21 (18.44)	0.002
Vitality	42.25 (23.91)	50.58 (24.54)	0.016
Social Aspects	27.38 (24.29)	62.08 (29.89)	<0.001
Emotional Aspects	32.00 (45.92)	57.37 (48.82)	<0.001
Mental Health	55.84 (24.58)	70.88 (21.71)	<0.001
Physical Health Dimension	26.24 (13.71)	41.61 (21.68)	<0.001
Mental Health Dimension	38.68 (19.52)	57.01 (21.30)	<0.001

* SD=standard deviations; Test used: Mann-Whitney

## Discussion

The predominance of the female gender (74.5%) among venous insufficiency patients, observed in this study, was reported by other authors^(^ 3 ^,^ 10 ^)^. In a study undertaken in Goiás, however, a higher predominance of VU was found in men^(^ 11 ^)^. As regards the age, this study shows the emergence of venous insufficiency characteristics, such as telangiectasias or spider veins, varicose veins, edema, lipodermatosclerosis as from the age of 18 years. The mean age of patients with venous ulcers was 60.6 years.

In a study undertaken in the Family Health Strategy (FHS) of a city in Goiás, the quality of life of patients with chronic lower limb injuries was assessed, showing that the patients' mean age was 62.7 years^(^ 6 ^)^. In another study in Maceió/AL, the predominance of CVD was identified in the age rage between 30 and 40 years among men and between 50 and 60 years among women^(^ 12 ^)^.

Professions similar to the patients in this study (domestic servant, driver, cashier, telephone operator, craftsman and cook) were found in other studies^(^ 13 ^-^ 15 ^)^, with a predominance of professions that demand little mobility, long periods in orthostatic positions and short rest times, which can be a risk factor to trigger venous hypertension in the lower limbs, besides the emergence and chronic nature of VU.

The precarious income and low education levels observed in the study sample are constant factors in venous disease patients^(^ 3 ^,^ 6 ^,^ 13 ^,^ 16 ^)^, which can indicate a lifestyle that favors the appearance of injuries or the lack of access to specialized health services. In a loss-making economic situation, the presence of the wound and the care it demands are a destabilizing factor in the family's financial balance^(^ 17 ^)^.

The relation between chronic conditions and the presence of venous ulcer has been analyzed, showing higher frequencies among patients with VU (60.0%), confirming reports by other authors. In a study undertaken in the Family Health Strategy in a city in Goiás^(^ 6 ^)^, 21.2% of hypertensive people were found among patients with chronic lower limb injuries. A study involving VU patients^(^ 11 ^)^ observed that 36.2% suffered from hypertension and 17.2% from diabetes. In another study^(^ 3 ^)^ that analyzed 50 patients with CVD, 26 manifested associated chronic conditions, the most frequent of which was arterial hypertension.

Disturbed sleep pattern is an experience VU patients report, almost always associated with pain^(^ 17 ^-^ 19 ^)^. In this study, however, most participants (77.0%) slept a satisfactory number of hours.

In this study, the QoL of venous disease and ulcer patients was lower in all domains when compared to patients without VU, confirming the results of other studies in which the most advanced stage of the venous disease showed a significant impact of the quality of life of VU patients^(^ 3 ^,^ 5 ^,^ 7 ^)^.

The loss observed in these patients' quality of life was mainly related with the physical aspect and functional ability, domains that showed the lowest scores, confirming the results of another study^(^ 3 ^)^. In fact, the presence of the ulcer affects individuals' perception of their physical wellbeing and limits activities of daily living and professional activities. Routine activities like climbing or moving down stairs or simply standing without support for a short period become difficult to accomplish in daily life. The interference in locomotion entails multiple limitations, obliging people with VU to restructure their daily activities and, in some cases, to feel dependent on others, also hampering the social relations^(^ 17 ^)^.

The presence of venous ulcer can make the patients feel socially isolated, depressed and constrained due to the dressings, as these patients generally have their legs bandaged, making them feel ashamed to get close to other people, which can make it difficult to maintain and increase their social cycle of friendships^(^ 15 ^)^. Other studies report on the social impact of VU, showing that the patients feel discriminated against by their family, society and even themselves^(^ 6 ^,^ 12 ^)^.

The low QoL scores observed in the domains emotional aspect and mental health among VU patients underline the fact that the presence of the ulcer also affects these patients' mental health. In a literature review that analyzed the quality of life of patients with venous ulcers, it was concluded that the aspects in the physical and mental health dimensions, such as sadness due to altered bodily image, physical limitation and pain, are frequent in QoL assessment studies of patients with venous ulcer^(^ 20 ^)^.

Studies show the presence of pain as a factor that provokes great discomfort, besides limiting the activities of daily living^(^ 5 ^,^ 20 ^)^. In the same sense, in a study by nurses in the United Kingdom, it was verified that the participants described the pain as a constant reminder of their ulcer, that it was tireless and contributed to their feelings of loss of control. The authors found that the pain was related to the loss of mobility, sleep disorders, negative psychological effect and reduced quality of life^(^ 18 ^-^ 19 ^)^.

This study comes with some limitations that should be observed. The medication therapy was not assessed and this variable could interfere in the patients' perceived quality of life. Nevertheless, all patients were attended by the same medical team and may have received similar pharmacological interventions. In the education categories, illiterate subjects were grouped with individuals who had finished primary education, and this decision may have included very different subjects in the same category. These limitations should be overcome in future studies.

## Conclusion

Patients with CVD and venous ulcer showed significant impairments in their quality of life when compared to patients with CVD without venous ulcer. The quality of life aspects the presence of ulcers affected most were: physical aspect, functional capacity, social aspects and physical health.

In view of the impact of the venous ulcer in the patients' quality of life, it is important for the nurses to heed the evolution of the CVD in the attempt to prevent ulcers and appropriately treat the cases of existing ulcers, with a view to minimizing the impairments these patients may suffer in their quality of life.
